# Can irrelevant self‐related information in working memory be actively suppressed?

**DOI:** 10.1002/pchj.790

**Published:** 2024-07-31

**Authors:** Quan Zhang, Tiangang Zhou, Jin Tang, Huanjun Xi

**Affiliations:** ^1^ School of Mental Health and Psychological Sciences Anhui Medical University Hefei China; ^2^ Institute of Artificial Intelligence Hefei Comprehensive National Science Center Hefei China; ^3^ State Key Laboratory of Brain and Cognitive Science, Institute of Biophysics Chinese Academy of Sciences Beijing China; ^4^ University of Chinese Academy of Sciences Beijing China

**Keywords:** attended but outdated, inhibition effect, self‐associated, working memory

## Abstract

To utilize the resource of working memory efficiently, the brain actively suppresses irrelevant information to focus cognitive resources on the task at hand. However, whether task‐irrelevant self‐related information can be suppressed is still an open question. This study explores the inhibitory effects of various types of identity‐associated information (self, friend, stranger) with an irrelevant distracting paradigm, in which participants are required to memorize the color while ignoring the shape during a memory array. In the subsequent test array, participants are asked to judge whether the color of the test item is the same as the memorized one, while the ignored shape features could also change. The results are as follows. (1) Self‐associated information survived the inhibitory effect no matter whether the interstimulus interval (ISI) was short or long. (2) Stranger‐associated information remained inhibitory effect in a long ISI (3000 ms). The results indicate that self‐associated information can bypass the executive system and remain active in working memory processing.

## INTRODUCTION

The concept of self plays a critical role in understandings of human higher‐level cognitive activities. Researchers have observed that self‐information can elicit faster responses compared with other‐information when using stimuli such as self‐face and name (Alexopoulos et al., 2012; Keyes & Brady, 2010; Liu et al., 2016). Sui et al. (2012) introduced the concept of the self‐association paradigm, which requires participants to associate identity labels (self, friend, and stranger) with simple geometric shapes (circle, square, and triangle) and to complete an associative‐learning task. This paradigm provided well‐controlled stimulus materials and reduced the influence of the familiarity factor compared with studies using one's own name or face. Over the past decade, this paradigm has been widely applied to investigate self‐referential advantages at various cognitive stages, such as perception (Sui et al., 2012; Sui & Humphreys, 2015a), attention (Macrae et al., 2018), and decision‐making (Stolte et al., 2017). In addition, a recent study (Yin et al., 2019) has documented an automatic prioritization of self‐referential items in visual working memory (WM).

WM is an online temporary storage system that involves the manipulation of information during cognitive activities (Baddeley, 2003). It is believed to be central to human cognition. The capacity limitations of WM necessitate a balance between stability (maintaining and inhibiting) and flexibility (updating and clearing) (Trutti et al., 2021). Attention is considered to act as the “gateway” to WM, because it determines which information enters WM (Awh et al., 2006; Gazzaley & Nobre, 2012). This means that more attention can promote memory (Ravizza & Hazeltine, 2013). In addition to needing to maintain goal‐relevant information for decision‐making, humans often encounter attended but outdated information (referred to as key features), such as checking target images in human–machine verification. During verification, individuals need to keep their attention on identifying whether the image is the required target, but once the verification is successfully finished, they have difficulty recalling the target image. Fu et al. (2021) showed that to prevent interference with subsequent task representation, attended but outdated information (key features) entering WM, though highly activated, is actively suppressed by the executive control system. Their results also showed that the inhibition effect of key features takes a relatively long encoding time. Surprisingly, Fu et al.'s study enriches our understanding of the relationship between WM and attention.

Attention‐processing mechanisms are generally categorized into two types: stimulus‐driven attention and goal‐driven attention (Yantis, 2000). Goal‐driven attention refers to the allocation and regulation of attention resources to achieve task‐related goals. In contrast, stimulus‐driven attention involves the automatic capture of attention by salient features. On the one hand, compared with other irrelevant information, self‐related information, owing to its high social salience, has the capacity to capture attention in a bottom‐up manner. On the other hand, researchers have found that irrelevant information encoded in WM can divert attention from the current task through top‐down attention guidance (Carlisle & Woodman, 2011; Downing, 2000; Kumar et al., 2009). It suggests that irrelevant self‐information encoded in WM attracts stronger attention on both channels. Therefore, whether this attended self‐information can be suppressed by WM when it is also task‐irrelevant remains unclear, which is essential for performing cognitive activities correctly and smoothly.

Based on the theory of Fu et al. (2021) that attended but outdated (high‐activation) information is inhibited by WM, our research aims to determine whether social attributes (especially self‐related) as highly activated information are also inhibited. Thus our study employs an experimental design with key features, which is achieved by attaching social labels to shapes in the irrelevant distracting paradigm.

The irrelevant distracting paradigm is a classic paradigm for studying the relationship between attention and WM (Ecker et al., 2013; Gao et al., 2011). The experiment sequentially presents a memory array and a test array. Participants must determine whether a specific feature dimension (referred to as the response feature) has altered in the test array compared with in the memory array, while disregarding changes in other feature dimensions that may also occur. Changes in these ignored features can negatively impact the ability to detect changes in the response feature, thereby creating an effect known as irrelevant‐change distraction. Fu and colleagues' study (Fu et al., 2021) indicates that the interference effect disappears owing to the suppression of key features. Therefore, we use the size of the interference effect as an indicator to verify whether social attribute information can be inhibited by WM.

We assumed that self‐related information maintained in WM cannot be actively suppressed. In Experiment 1, we used geometric shapes (circle, triangle, square) associated with identity labels (self, friend, stranger) as key features, and required participants to complete a classical irrelevant‐distracting task (Fu et al., 2021; Hyun et al., 2009). In Experiment 2, we investigated whether the WM maintenance time is the key factor of the inhibition effect by manipulating the time of the interstimulus interval (ISI) between the memory array and test array. To preview our results, the present study provides converging evidence that attended but outdated self‐related features cannot be inhibited by the brain.

## EXPERIMENT 1

### Participants

To replicate the self‐advantage effect in the WM task, we used G*Power 3.1.9 (Universitat Kiel, Kiel, Germany; Faul et al., 2007) to estimate the effect size *η* ^2^ from previous research (Yin et al., 2019) as 0.5. To detect an effect with a power of 0.80 at a significance level of *α* = 0.05, a minimum sample size of 24 participants was required. In this study, we recruited a total of 46 undergraduate students (33 females, *M*_age_ = 19.69 ± 1.98). All participants were right‐handed, reported normal or corrected‐to‐normal vision, and had no color blindness. The study was approved by the Ethics Committee of Anhui Medical University, Anhui Province, China.

### Apparatus and materials

**FIGURE A1:**
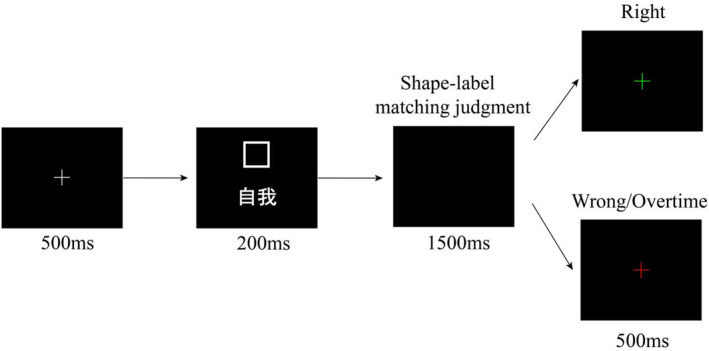
Procedure of the associative‐learning task.

**FIGURE A2:**
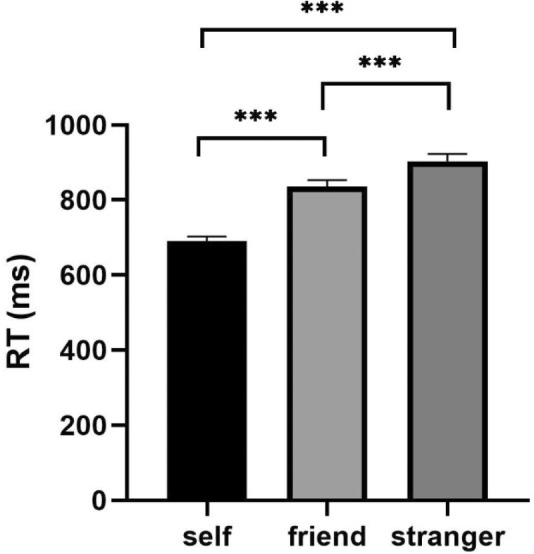
Reaction time (RT) as a function of label (self, friend, and stranger) in matching trials. The error bars represent the standard error; ***p < .001.

**FIGURE A3:**
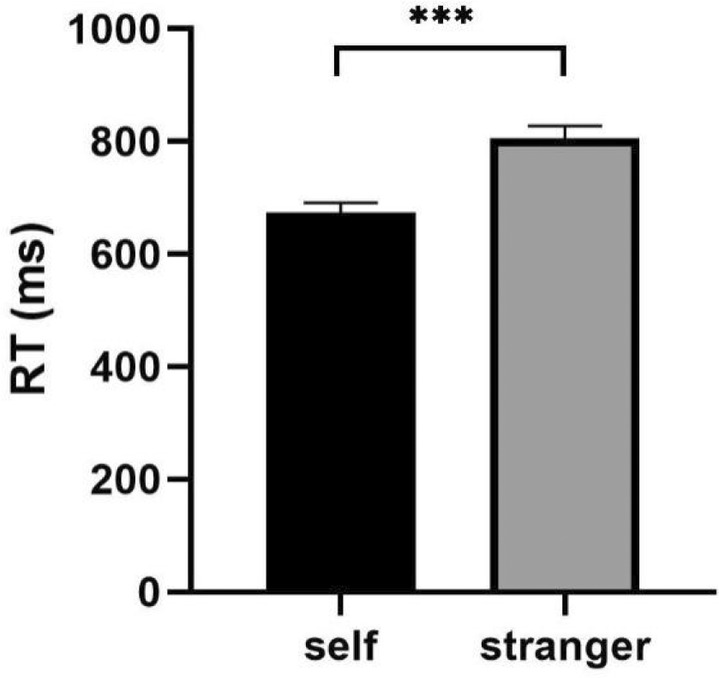
Reaction time (RT) as a function of label (self, stranger) in matching trials. The error bars represent standard error; *** *p* < .001.

The experiment was programmed using Psychtoolbox (University of California, Santa Barbara, CA, USA; Brainard & Vision, 1997; Kleiner et al., 2007) in (MATLAB, MathWorks, Natick, MA, USA; 2021a). The stimuli were presented on a 15.6‐in. screen (1920 × 1080, 60 Hz), with participants seated 90 cm away from the screen. In the irrelevant distracting task, labels were in black font, and geometric shapes were in three colors: red, green, and blue (see Figures A1, A2, A3). All stimuli were presented at the center of the white background screen, subtending a visual angle of 6 deg × 6 deg.

### Experimental design and procedure

The irrelevant distracting task was conducted as a 3 (labels: self, friend, stranger) × 2 (shape change: unchanged, changed) × 2 (color sameness: same, different) within‐subject design, with 36 trials per condition. To keep participants focusing on a shape–label matching task and paying attention to shapes, 36 catch trials (mismatch of shape–label pairs) were added, in which participants are not required to press a key response if the shape–label pairs did not match. The experiment was divided into six blocks, each including 72 trials and 6 catch trials. Before the formal experiment, participants completed 20 practice trials. Only those with an accuracy rate of 80% or higher proceeded to the formal experiment.

**FIGURE 1:**
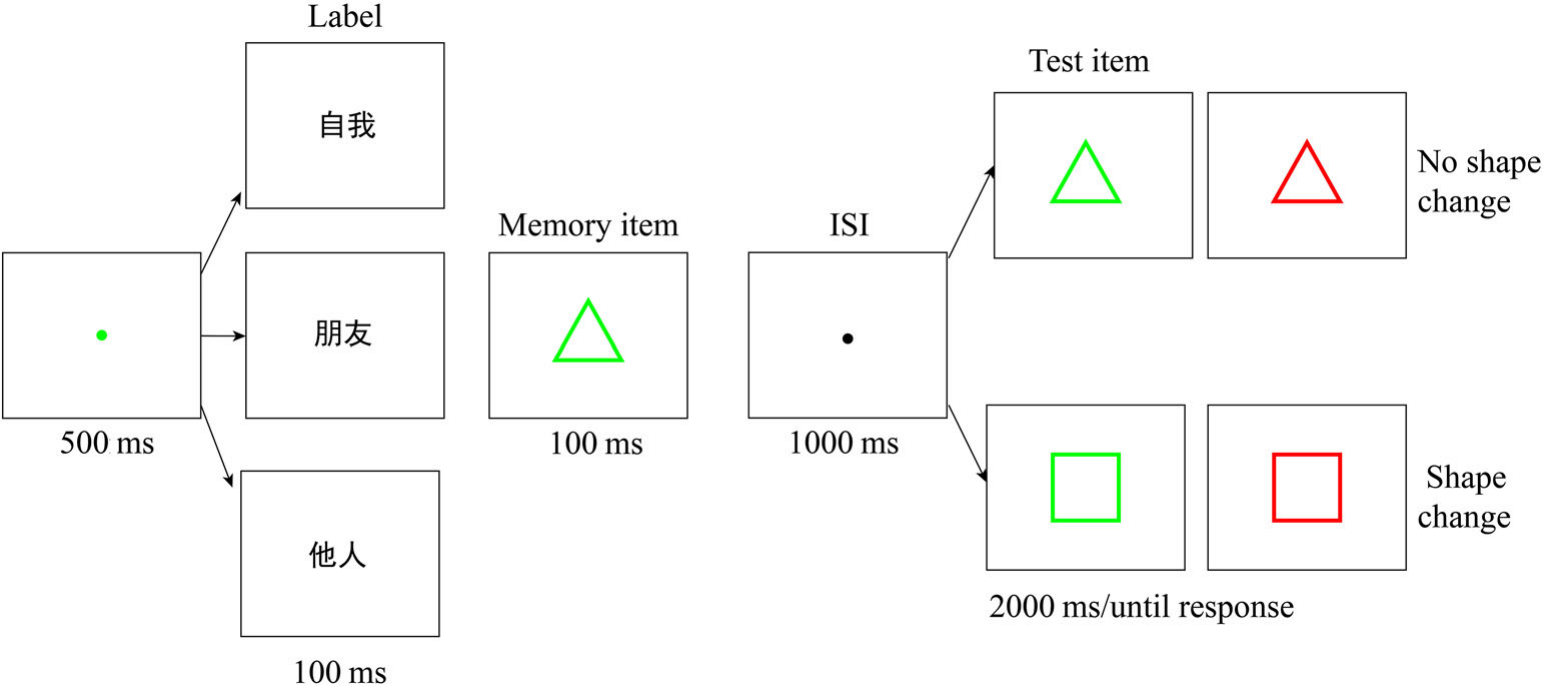
The procedure of the irrelevant distracting task in Experiment 1. Participants were required to remember the color of the memory item and complete a color‐change detection task in the test. In the memory maintenance stage, they needed to judge whether the shape of a memorized item matched the former identity label. If the shape–label pairs did not match, they held response and conducted the next trial. Labels from the top to the bottom are Self, Friend and Stranger. ISI = Interstimulus interval.

Experiments 1 and 2 consisted of two stages, a training phase (an associative‐learning task) followed by a test phase (an irrelevant distracting task). The procedure of the associative‐learning task (see Appendix A) was the same as in previous research (Sui et al., 2012). The irrelevant distracting task began with a green fixation point (0.05 deg × 0.05 deg, 500 ms) presented in the center of the white background screen, followed by a label (6 deg × 6 deg, 100 ms) and then a colored shape (6 deg × 6 deg, 100 ms), as shown in Figure 1. The colored shape was a memorized item and was maintained for 1000 ms. Participants were required to judge whether it matched with the former label. If the current trial was a catch trial (shape–label mismatch), participants were asked to make no response and no test array was presented. Otherwise, a test array was presented in the center of the screen for 2000 ms, in which the shape feature randomly changed compared with that in the memory array. During the test array, participants were required to press one of two keys if the color of the test item was the same as that of the memorized item. The assignment of the response key to “same” or “different” responses was counterbalanced across participants.

### Results and analysis

A total of 46 participants took part in the experiment, with four participants excluded from the analysis because of their poor performance in the catch trials. Responses with RTs of less than 200 ms and those more than three standard deviations from the mean RTs within each condition for each participant were removed. The mean reaction time (RT) and accuracy under each condition are shown in Table 1. The statistical analysis was conducted based on data from two tasks.

**TABLE 1:** Accuracy and mean reaction times (M ± SD) as a function of label (self, friend, stranger) and deformation (shape unchanged, shape changed) in Experiment 1.

Label	Shape change	Accuracy (%)	Mean RT (ms)
Self	Unhanged	93 ± 6.0	736 ± 101
	Changed	94 ± 5.4	777 ± 113
Friend	Unchanged	93 ± 5.9	760 ± 94
	Changed	92 ± 6.2	778 ± 109
Stranger	Unchanged	93 ± 6.1	768 ± 98
	Changed	91 ± 7.7	793 ± 104

Abbreviations: Accuracy = proportion correct; RT = reaction time.

**FIGURE 2:**
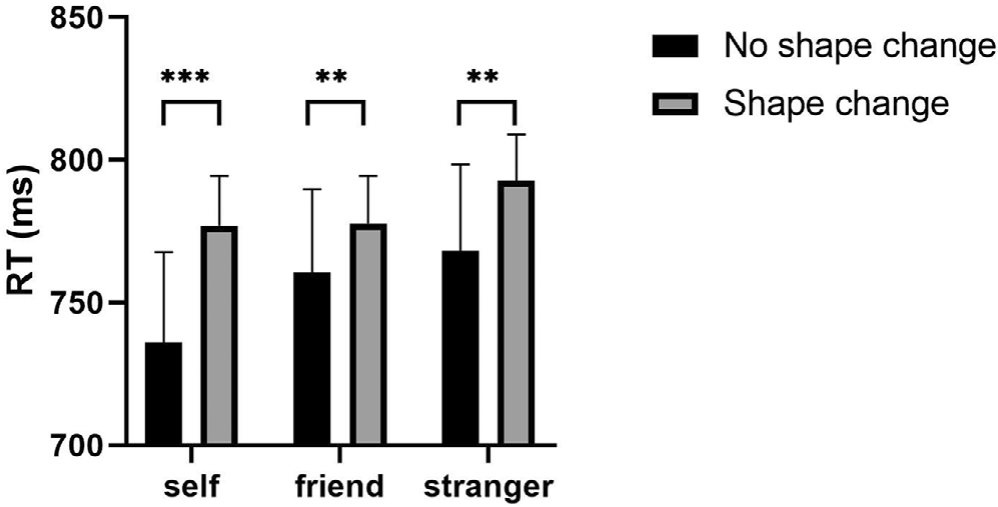
Reaction time (RT) as a function of label (self, friend, stranger) and deformation (shape unchanged, shape changed). The error bars represent the standard error; **p < .01; ***p < .001.

The results of the associative learning task were consistent with previous research (Sui et al., 2012; Yin et al., 2019). Statistical results indicate that both reaction times (RTs) and accuracy rates under self‐associated conditions were significantly better than those under other conditions (Accuracy: self–friend *p* < .001, self–stranger *p* = .001; RTs: self–friend *p* < .001, self–stranger *p* < .001). For detailed analysis, please refer to the Appendices. In the irrelevant distracting task, a repeated‐measures analysis of variance (ANOVA) was conducted on accuracy and RT data with factors of 3 (labels: self, friend, stranger) × 2 (shape change: unchanged, changed). The main effects of both the label [*F* (2, 82) = 7.360, *p* = .001, *η* ^2^ = 0.150] and the shape change [*F* (1, 41) = 28.969, *p* < .001, *η* ^2^ = 0.410] were significant, and post hoc comparisons revealed that RTs under the self condition were significantly faster than those under the stranger condition (*p* < .010), with no significant difference between the self and friend conditions (*p* = .101), nor between the friend and other conditions (*p* = .707). RTs in the shape‐unchanged condition were significantly faster than in the shape‐changed condition (*p* < .001). The interaction between the two factors was also significant [*F* (2, 82) = 3.442, *p* = .037, *η* ^2^ = 0.077]. Post hoc multiple comparisons showed that the self‐label condition was significantly faster than the friend‐label condition (*p* = .008) and the stranger‐label condition (*p* = .001) (see Figure 2 and Table 1). It is worth noting that RTs in unchanged trails were significantly faster than those in changed trials (*p*_self_ < .001, *p*_friend_ = .008, *p*_stranger_ = .003), suggesting that the shapes associated with social attributes, serving as a key feature, interfered with color judgment. Furthermore, the significant interaction effect suggested that the size of interference effects of different social attributes is not equal; that is, the interference effect under the self label is significantly greater than that under friend and stranger label (as shown in Figure 2), suggesting that in addition to the interference effect brought by social attributes, there is also an advantage effect of self relative to friend and stranger, which echoes results of the associative‐learning task. Hence, our results document a significant irrelevant‐change distracting effect when social labels serve as key features.

### Discussion

In the irrelevant distracting task, we found two significant results. First, for the condition of unchanged shapes, the RT of self label was significantly faster than that of the friend label and other label. This finding aligns with results in Yin et al. (2019), indicating a robust self‐prioritization effect in WM. On the one hand, we interpret this data as reflecting that a self‐associated shape maintained in WM can attract greater endogenous attention than an other‐associated shape (Yin et al., 2019). On the other hand, we argue that the integrative self hypothesis proposed by Sui and Humphreys (2015b) can help to explain our results. According to their theory, self‐referential can facilitate the integration of all the features to form a whole representation, thus leading to faster RTs for self‐associated representation than for other‐associated representation. Second, we found a significant irrelevant‐change distracting effect when key features were associated with social labels, which is contrary to the results in Fu et al. (2021). Although Fu and colleagues argued that the key feature was actively inhibited by the cognitive system during the maintenance phase of WM, they also acknowledged that the inhibition of a key feature was not easy, especially when it received high activation. The shapes associated with social labels may have obtained more attention resources during the shape‐label matching judgement, thus receiving higher activation. Therefore, one may argue that inhibition of key features takes time and the duration of WM maintenance may influence the inhibition effect. To test this assumption, we manipulated the ISI between the memory display and target display in Experiment 2.

## EXPERIMENT 2

### Experimental design and procedure

**FIGURE 3:**
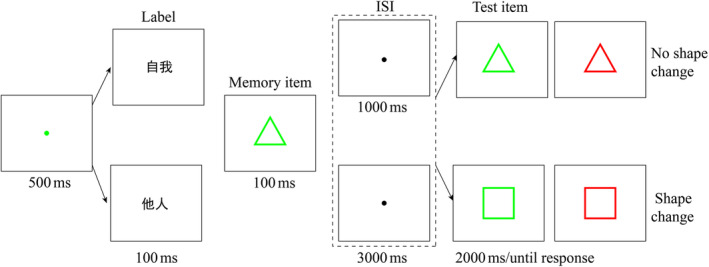
The procedure of the irrelevant distracting task in Experiment 2. On the basis of the Experiment 1 procedure, removing the friend label while keeping the self and the stranger labels, and setting the interval between memory item and test item as a variable (1000 ms/3000 ms).

According to a previous study (Fu et al., 2021), we examined the effect of the maintenance interval by setting the ISI level (1 and 3 s) between memory display and target display. The friend label, which resulted in the same outcome as the stranger label in Experiment 1, was removed from this experiment. Additionally, two neutral shapes, not associated with any labels, were introduced (see Figure 3 and Appendix A). The experiment design was the same as that in Experiment 1 except for the introduction of the factor of ISI level. The experiment was divided into six blocks, each consisting of 86 trials, with a 3‐min break between the blocks.

### Results and analysis

**FIGURE 4:**
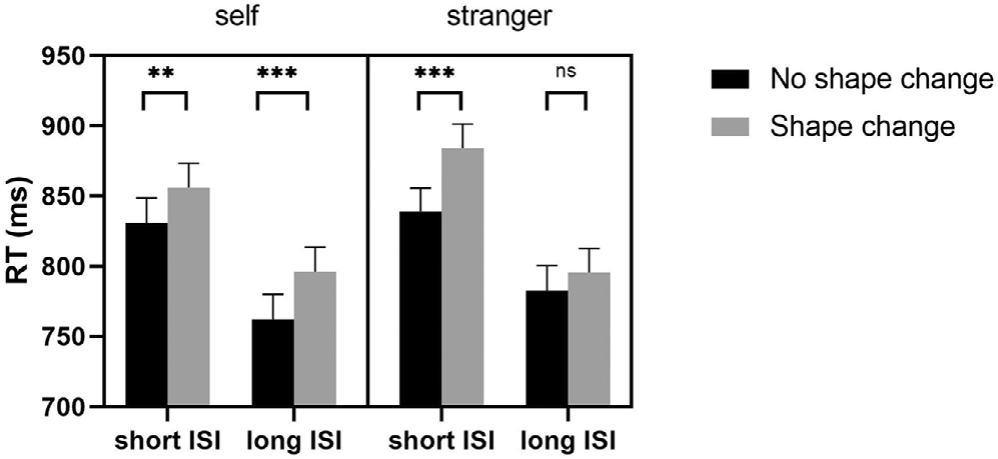
Reaction time (RT) as a function of interstimulus interval (ISI, short ISI: 1 s, Long ISI: 3 s), label (self, stranger), and deformation (shape unchanged, shape changed). The error bars represent the standard error; **p < .01; ***p < .001; ^ns^ *p* > .05.

Thirty‐eight participants took part in Experiment 2 (27 females, *M*_age_ = 20.22 ± 1.99), with two participants excluded from the analysis owing to poor performance in the catch trials. The results of the associative‐learning task were consistent with those in Experiment 1, as described in the Appendices (Accuracy: self–stranger *p* < .001; RTs: self–stranger *p* < .001). Data processing and exclusion criteria were the same as in Experiment 1. A repeated‐measures ANOVA was conducted on RT data with factors of 2 (labels: self, stranger) × 2 (shape change: unchanged, changed) × 2 (ISI: 1 s, 3 s). The main effects of the label [*F* (1, 35) = 12.939, *p* = .001, *η* ^2^ = 0.270], shape change [*F* (1, 35) = 47.391, *p* < .001, *η* ^2^ = 0.580], and ISI [*F* (1, 35) = 67.622, *p* < .001, *η* ^2^ = 0.660] were all significant. Furthermore, the three‐way interaction among label, shape change, and ISI interval was significant [*F* (1, 35) = 12.071, *p* = .001, *η* ^2^ = 0.260]. Post hoc multiple comparisons revealed significant main effects of both label (*p* = .010) and ISI (*p* < .001). The significant three‐way interaction indicates that there are different interference effects for different social attributes and different ISIs. Under the short‐ISI (1‐s) condition, post hoc multiple comparisons (Bonferroni‐corrected) showed that key features associated with self and stranger both had significantly faster RTs in the shape‐unchanged condition compared with the shape‐changed condition (*p*_self_ = .001, *p*_stranger_ < .001), which repeated the results of Experiment 1. However, under the long‐ISI (3‐s) condition, the RT of self‐association was significantly faster in the shape‐unchanged condition than in the change condition (*p* < .001), and there was no significant difference between the unchanged and changed conditions for stranger association, indicating an irrelevant‐change distracting effect of key features associated with self (see Figure 4 and Table 2).

**TABLE 2:** Accuracy and mean reaction times (M ± SD) as a function of label (self, stranger), interstimulus interval (1 s, 3 s) and deformation (shape unchanged, shape changed) in Experiment 2.

Label	ISI	Shape change	Accuracy (%)	Mean RT (ms)
Self	1 s	Unchanged	95 ± 4.2	831 ± 107
		Changed	93 ± 5.7	856 ± 104
	3 s	Unchanged	96 ± 3.8	762 ± 106
		Changed	96 ± 4.1	796 ± 104
Stranger	1 s	Unchanged	94 ± 4.8	839 ± 101
		Changed	92 ± 6.0	884 ± 105
	3 s	Unchanged	97 ± 4.3	783 ± 107
		Changed	96 ± 3.9	795 ± 105

Abbreviations: Accuracy = proportion correct; ISI = interstimulus interval; RT = reaction time.

### Discussion

The results of the irrelevant distracting task confirmed the hypothesis that the maintenance interval of WM exerted an influence on the inhibitory effect of key features. When provided with a sufficient maintenance interval (3 s), key features associated with the stranger label exhibited an inhibitory effect and no longer interfered with color judgments. However, when self label was attached to key features, it still divorced from inhibition of WM and affected the judgement of the subsequent color task. We interpret this data as reflecting an object‐based representation of the self‐related item in WM. In common with the idea that a self‐related stimulus is a powerful exogenous cue and captures exogenous attention (Humphreys & Sui, 2016; Liu et al., 2016; Sui & Rotshtein, 2019), a self‐related stimulus it can also attract internal attention during the memory maintenance phase to keep the representation activated (Yin et al., 2019). As with object‐based attention, integrated object representations of WM state that memory stores integrated objects, not independent features. Owing to the association with the self label, the key features gained attention and were represented in a coherent object, which led to a faster response in the unchanged condition than in the changed condition.

## GENERAL DISCUSSION

This study has provided behavioral evidence to answer the question of whether irrelevant self‐related information in WM can be actively inhibited. We conducted two experiments with an irrelevant distracting task in which label‐associated (self, friend, and stranger) shapes were used as key features. In Experiment 1, we found a significant irrelevant‐change distracting effect when key features were associated with social labels. In Experiment 2, we manipulated the maintenance interval of WM by setting different ISI levels between the memory display and the target display. The results indicated that self‐associated key features manifested a robust irrelevant distracting effect, no matter whether the ISI was short or long. However, the inhibition of stranger‐associated key features was dependent on the maintenance interval. Based on our behavioral data, we have shown that self‐related information is prioritized over other‐related information in WM, thus making it difficult to actively inhibit the former.

We propose two hypotheses to explain why self‐related information survived the inhibition of WM. First, according to the integrative self‐account proposed by Sui and Humphreys (2015b), self‐reference plays a role in the integration of parts into perceptual wholes and in binding together different types of information. Hence, self‐associated key features facilitate the memory of self‐related items, thus exhibiting an irrelevant‐change distracting effect on the color task. Although the key features are irrelevant shapes, self‐reference integrates shape and other features (i.e., color) into a whole object representation, so the cognitive system fails to suppress a single shape feature. The second hypothesis is based on object‐based representations in WM (Cowan, 2001; Luck & Vogel, 1997; Zhang & Luck, 2008). This object representation is characterized by objects being encoded holistically and irrelevant features also being represented. This object‐based representation may be achieved through attention, as attention is attracted to self‐related items internally during the WM maintenance. Therefore, key features associated with self prioritize others and affect the subsequent color task. These two explanations may not be mutually exclusive, and it is possible that both work at the same time. Future studies that focus on object‐based self‐representation may help us better understand the relationship between attention and WM.

The inhibitory effects of key features associated with the stranger label suggest that the maintenance time of information (whether it is outdated or not) plays a moderating role between attention and WM. If outdated information receives more attention (high activation), it not only fails to be reinforced in subsequent WM, but also is actively suppressed to ensure that the target information is protected from interference. Our results demonstrate that maintaining the high activation of different social attributes requires different ISIs. Self‐referential information, owing to its higher activation, may require a longer maintenance time or may not be suppressible at all, whereas for friend‐ or other‐referential information, owing to the limitations of WM capacity, high activation can be maintained only for a shorter period. In summary, our results indicate a special representation mechanism for self‐related information in WM.

Our results suggest that self‐representation maintained in WM breaks the balance between stability and flexibility. What makes self‐related information special in WM? We argue this may be a quasi‐automatic function of the self‐referential system of the brain. When new self‐related information enters WM, the self‐referential system preserves the self‐representation to decide whether to internalize it as part of the self or to eliminate it. The neural mechanism of the self‐referential function may be related to the ventral medial prefrontal cortex (VMPFC). The VMPFC is a well‐established brain region that involves self‐related processing (Northoff et al., 2006; Sui et al., 2013; Sui & Gu, 2017). A previous study (Yin et al., 2021) demonstrated that maintenance of self‐representation may occur through enhanced functional connectivity of the VMPFC to the WM network (e.g., in the superior parietal lobule). In addition, interactions between the VMPFC and top‐down cognitive control (e.g., in the dorsolateral prefrontal cortex) may shed light on understanding why self‐associated key features cannot be suppressed by the brain.

## CONCLUSION

In conclusion, this study shows that attended but outdated self‐associated information distracts an ongoing task, no matter how long the maintenance interval is. Particularly, we assume that the self‐representation maintained in WM is based on a holistic object, which integrates irrelevant features into a whole object and remains highly activated. The present study deepens our understanding of the relationship between attention and WM and provides new insights into the cognitive mechanisms of self‐representation during the WM maintenance stage.

## FUNDING INFORMATION

This work was supported by the National Science and Technology Innovation 2030 Major Program (2021ZD0203803) and the Ministry of Science and Technology of China (2020AAA0105601).

## CONFLICT OF INTEREST STATEMENT

The research was conducted in the absence of any commercial or financial relationships that could be construed as a potential conflict of interest.

## ETHICS STATEMENT

Approval was obtained from the Ethics Committee of Anhui Medical University. The procedures used in this study adhere to the tenets of the Declaration of Helsinki.

## CONSENT TO PARTICIPATE

Informed consent was obtained from all individual participants included in the study.

## CONSENT FOR PUBLICATION

Participates signed informed consent forms regarding publishing their data and photographs.

## Figures and Tables

**FIGURE A4:**
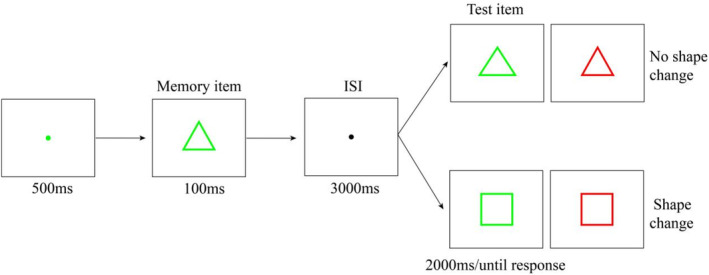
The procedure of the irrelevant distracting task.

**FIGURE A5:**
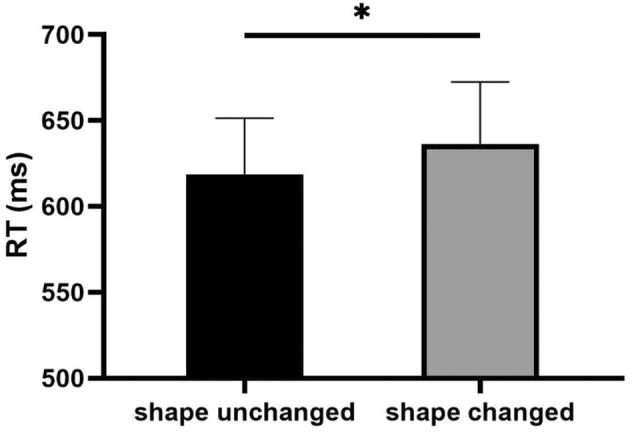
Reaction time as a function of shape change in the color judgment task. Error bars represent standard error, and * means *p* < .05.

## Data Availability

The datasets generated during the current study and experiment materials are available at https://osf.io/79zkp/.

## APPENDIX A

### METHOD FOR ASSOCIATIVE‐LEARNING TASK

In the associative‐learning task, white geometric shapes (circle, triangle, square, 2.4 deg × 2.8 deg) were presented above (2.3 deg) the center of the black background screen, with the Chinese character identity labels (self, friend, stranger, 3 deg × 2.8 deg) displayed below (2.3 deg). The associative‐learning task was conducted as a 3 (labels: self, friend, stranger) × 2 (match: match, mismatch) within‐subject design, with 36 trials per condition. The experiment was divided into two blocks, each consisting of 108 trials, with a 3‐min break between the blocks. Before the formal experiment, participants completed 10 practice trials. Only those with an accuracy rate of 80% or higher proceeded to the formal experiment. The procedure of the associative‐learning task (see Figure A1) is the same as in previous research (Sui et al., 2012).

### EXPERIMENT 1 RESULTS FOR THE ASSOCIATIVE‐LEARNING TASK

For the associative‐learning task, previous research has shown that the self‐referencing effect is more pronounced under matching conditions. Therefore, we conducted a repeated‐measures ANOVA for reaction time (RT) data and accuracy data, separately.

#### Accuracy

The main effect of identity labels was significant [*F* (2, 40) = 13.804, *p* < 0.001, *η* ^2^ = 0.41]. Post hoc multiple comparisons revealed that accuracy under the self‐label condition was significantly higher than that under the stranger‐label condition (*p* < .001), and accuracy under the friend‐label condition was also significantly higher than that under the stranger‐label condition (*p* = .001). There was no significant difference between the self‐label and friend‐label conditions.

#### RT

We excluded RTs below 200 ms and data beyond three standard deviations from the mean of correct responses. The analysis showed a significant main effect of identity labels [*F* (2, 82) = 77.484, *p* < .001, *η* ^2^ = 0.65; see Figure A2, Table A1]. Post hoc multiple comparisons revealed that RT under the self‐label condition was significantly faster than that under the friend‐label condition (*p* < .001) and the stranger‐label condition (*p* < .001). The friend‐label condition was also significantly faster than the stranger‐label condition (*p* = .001).

**TABLE A1:** Accuracy and mean reaction time (M ± SD) as a function of label (self, friend, and stranger) in the matching trial.

Label	Accuracy (%)	RT (ms)
Self	97 ± 5.1	691 ± 74
Friend	95 ± 4.0	836 ± 105
Stranger	90 ± 8.7	904 ± 124

Abbreviations: Accuracy = proportion correct; RT = reaction time.

### EXPERIMENT 2 RESULTS FOR THE ASSOCIATIVE‐LEARNING TASK

Consistent with the Experiment 1, we conducted paired‐sample *t* tests on response times and accuracy for different labels under matching conditions.

The results indicate that the accuracy of self‐associated trials is significantly higher than the accuracy of stranger‐associated trials (*t*_35_ = 4.535, *p* < .001, Cohen's *d* = 0.898). For the RT data, we excluded RTs below 200 ms and data beyond three standard deviations from the mean of correct responses. The analysis showed a significant time advantage for self‐associated trials (*t*_35_ = −9.777, *p* < .001, Cohen's *d* = −1.089) (Table A2).

**TABLE A2:** Accuracy and mean reaction times (M ± SD) as a function of label (self, stranger) in matching trials.

Label	Accuracy (%)	RT (ms)
Self	98 ± 2.9	673 ± 108
Stranger	93 ± 5.9	805 ± 133

Abbreviations: Accuracy = proportion correct; RT = reaction time.

### EXPERIMENT 1 ACCURACY RESULTS FOR THE IRRELEVANT DISTRACTING TASK

A repeated‐measures analysis of variance (ANOVA) was conducted on accuracy data with factors of 3 (label: self, friend, stranger) × 2 (shape change: unchanged, changed). The main effect of label was significant [*F* (2, 82) = 3.808, *p* = .026, *η* ^2^ = 0.08], and the interaction between label and shape change was significant [*F* (2, 82) = 3.346, *p* = .04, *η* ^2^ = 0.08]. Post hoc multiple comparisons revealed that under the condition of shape changed, the self label had a significantly higher accuracy than the stranger label in matching trials (*p* = .007).

### EXPERIMENT 2 ACCURACY RESULTS FOR THE IRRELEVANT DISTRACTING TASK

A repeated‐measures ANOVA was conducted on accuracy data with factors of 2 (labels: self, stranger) × 2 (shape change: unchanged, changed) × 2 (ISI: 1 s, 3 s). The main effects of both shape change [*F* (1, 35) = 14.046, *p* = .001, *η* ^2^ = 0.29] and ISI [*F* (1, 35) = 31.949, *p* < .001, *η* ^2^ = 0.48] were significant, while the main effect of label and all interactions were not significant. Simple main effects analysis showed that accuracy under the shape‐unchanged condition was significantly higher than that under the shape‐changed condition (*p* = .001), and accuracy at the ISI of 3 s was significantly higher than that at the ISI of 1 s (*p* < .001).

### EXPERIMENT 3 ACCURACY RESULTS FOR THE IRRELEVANT DISTRACTING TASK

#### Objective

To verify whether the interference of shape change on color judgment disappeared because of active inhibition or self‐decay.

#### Participants

The experiment recruited 12 participants.

#### Apparatus and stimuli

The experimental materials were consistent with the main experiment.

Design and Procedure: The irrelevant‐feature interference task was designed as a 2 (shape: changed, unchanged) × 2 (color: changed, unchanged) within‐subjects design, with 36 trials per condition, totaling 144 trials. These were divided into two groups of 72 trials each, with a 3‐min rest interval between. Before the official experiment, participants were required to complete 10 practice trials, and only those who achieved an accuracy rate greater than 80% proceeded to the main experiment. As depicted in Figure A4, the experiment began with the presentation of a green fixation point on a white background screen, lasting for 500 ms, followed by a randomly colored and shaped figure for 100 ms. In both parts of the experiment, participants were instructed to remember the color of the shape. After the shape disappeared, a 3‐s blank screen with a black fixation point appeared. After 3 s, a shape of any color and form reappeared in the center of the screen. Participants were required to press a key as quickly as possible to determine whether the color of the shape matched the color they had memorized.

#### Results

RT and accuracy data for the irrelevant‐feature interference experiment are shown in Table A3 under the four conditions. In this part of the study, although color change judgment was the target task for participants, the experimental objective was to observe the impact of shape change on color judgment. Therefore, we combined the color‐change conditions and conducted a paired‐samples *t* test on the RTs of correct trials under the shape‐changed and shape‐unchanged conditions. The results showed that RTs were significantly faster under the shape‐unchanged condition than under the shape‐changed condition, indicating that shape changes interfered with color judgment even though the purpose of the shape was not explained before the experiment (shape unchanged: 618 ms, shape changed: 636 ms; *t*(11) = −2.273, *p* < .05, Cohen's *d* = −1.371) (Figure A5).

**TABLE A3:** Accuracy and mean reaction times (M ± SD) as a function of shape change in the color judgment task.

Shape	Color	Accuracy (%)	RT (ms)
Same shape	Same color	95 ± 3.3	582 ± 106
Same shape	Diff color	97 ± 3.1	615 ± 112
Diff shape	Same color	97 ± 3.2	655 ± 131
Diff shape	Diff color	97 ± 3.7	657 ± 146

Abbreviations: Accuracy = proportion correct; RT = reaction time.

### EXPERIMENT MATERIALS

Stimulus materials in Experiment 1 and Experiment 2 can be obtained at https://osf.io/79zkp/.
